# Vacunación contra COVID-19 y su afrontamiento desde la perspectiva de tres pueblos originarios de la sierra nororiental de Puebla, México

**DOI:** 10.1016/j.aprim.2022.102316

**Published:** 2022-02-17

**Authors:** Nancy Marbella Parra-Torres, Gildardo Bautista-Hernández, Abigail Techalotzi-Amador, Maylin Almonte-Becerril

**Affiliations:** División de Ciencias de la Salud, Universidad Intercultural del Estado de Puebla, México

Las enfermedades virales continúan emergiendo y representan un serio problema de salud pública^1^, presentando un impacto mayor en pueblos originarios. Ante esta situación, el objetivo de este proyecto fue conocer la percepción que presenta la población indígena perteneciente a 3 comunidades de la sierra nororiental del Estado de Puebla (Xonalpu, Ixtepec y Huehuetla), sobre la vacunación, y la práctica de medidas preventivas contra la COVID-19. El estudio se llevó a cabo en una muestra conformada por 246 participantes a los que se realizó una entrevista que incluyó 32 preguntas semiestructuradas.

Se evaluaron las características sociodemográficas presentes en la población, donde se tuvo un promedio de edad de 49 años, la mayoría era hablante de *tutunakú* y más del 80% pertenecía al nivel socioeconómico bajo o muy bajo. Los resultados concuerdan con los datos reportados en el plan de desarrollo regional de Puebla, que indica que en Huehuetla el 60,98% de la población vive en situación de pobreza, siendo, además, la localidad con mayor porcentaje de indígenas analfabetas (23,8%)^2^. Asimismo, aunque menos del 70% de los participantes recibieron información relacionada con la infección por COVID-19, esta fue impartida en castellano; factor que en otros estudios ya se ha comprobado que limita significativamente la adquisición efectiva de información entre el personal de salud y la población y que, en nuestros resultados, se asoció significativamente con no estar inmunizado (X^2^ = 10.910, p≤0,001). En cuanto a la vacunación, solo el 59,2% de los participantes recibieron la inmunización completa y solo el 45,3% de los participantes conocía el nombre de la vacuna que se le aplicó. Cuando se preguntó sobre las razones para no vacunarse, el 56,4% de los participantes no lo hizo por miedo, o porque no llegó la vacuna a su localidad (tabla 1).

**Tabla 1:** Clasificación por comunidad de las características sociodemográficas de la población participante (N = 246)

Variables	Municipio					
	Ixtepec		Xonalpu		Huehuetla	
	*(f)*	%	*(f)*	%	*(f)*	%
*Sexo*						
Masculino	21	31,3	20	34,5	39	32,2
Femenino	46	68,7	38	65,5	82	67,8
*Lengua*						
*Tutunakú*	63	94,0	55	94,8	105	86,8
Español	4	6,0	3	5,2	16	13,2
*Escolaridad*						
Sin estudios	21	31,3	20	34,5	37	30,6
Básico	35	52,2	29	50,0	69	58,0
Técnico	11	16,4	9	15,5	15	12,4
*Nivel socioeconómico*						
Alto	6,9	10,4	1,9	3,4	3,9	3,3
Medio	9,9	14,9	3,0	5,2	12,9	10,7
Bajo	11,9	17,9	8	13,8	15	12,4
Muy bajo	37,9	56,7	45	77,6	89	73,6
*Información sobre COVID-19*						
*¿Recibió información sobre COVID?*						
No	18	26,9	15	25,9	47	38,8
En castellano	49	73,1	43	74,1	74	61,2
*¿Le aplicaron la vacuna contra COVID?*						
No	15	27,9	27	46,6	47	39,2
Incompleto	5	7,5	3	5,2	2	1,7
Sí	47	70,1	28	48,3	71	59,2
*¿Por qué no se vacunó?*						
No quise/tuve miedo	12	60	12	40	34	69,3
No me enteré/ no avisaron	6	30	3	10	10	20,4
No llegó	2	10	15	50	5	10,2
*¿Qué es la COVID-19?*						
No existe/algo que mata	35	52,2	30	51,7	72	60
Un virus	32	47,8	28	48,3	48	40
*¿Usa cubrebocas?*						
No	31	46,2	12	20,6	30	24,7
Sí	36	53,7	46	79,4	91	75,3

Además, el contar con la inmunización se asoció con el uso de cubrebocas (X^2^ = 5,53, p≤0,001), el conocimiento y el nivel de estudios (X^2^ = 17,96, p≤0,001). En este sentido, es bien sabido que el éxito de las políticas para erradicar la COVID-19 depende de la aceptación que tengan las vacunas entre la población; dado que se requiere que al menos un 70% de la población esté vacunada, con el fin de detener los contagios y proteger a todas aquellas personas que no se pueden vacunar^3^. No obstante, las desigualdades estructurales y los determinantes sociales de la salud colocan a las poblaciones desfavorecidas en un mayor riesgo de contagio y muerte. Un estudio realizado en adultos jóvenes encontró que las diferencias raciales^4^ y la condición socioeconómica; así como el acceso limitado a la atención médica, influyen en la percepción sobre la vacunación^5^. Aunado a lo anterior, la tasa de prevalencia y letalidad de la COVID-19 resulta significativamente más elevada en pueblos originarios que en el resto de la población^6^, asociado a la estigmatización generada por los medios de comunicación y a la percepción de los riesgos asociados con las vacunas. Lo anterior ha favorecido que las poblaciones marginadas eviten acudir a las inmunizaciones, incrementando su susceptibilidad a la adquisición y propagación de la infección. Por tal motivo, puede decirse que las barreras geográficas, lingüísticas, económicas y culturales, han limitado significativamente la adquisición de conocimiento sobre la infección por COVID-19; así como la práctica de métodos preventivos en comunidades originarias y la disponibilidad a la inmunización. Por tanto, es necesario recurrir al diseño de estrategias de promoción de la salud que muestren una pertinencia cultural, a fin de generar una conciencia de prevención y mejora de los servicios de salud que permitan reducir al máximo la propagación de la infección y los efectos perjudiciales que esto conlleva.

## Financiación

Esta investigación ha sido financiada por el proyecto para el fortalecimiento de cuerpos académicos, a través del Programa para el Desarrollo Profesional Docente (PRODEP), para el Tipo Superior con el número UIEP-CA-07.

## Conflictos de interés

Todos los autores declaran no tener ningún conflicto de interés.
